# On the External Uses of Hydrate of Chloral

**Published:** 1876-11

**Authors:** William Craig


### ON THE EXTERNAL USES OF HYDRATE OF CHLORAL.

By Dr. WILLIAM CRAIG, F. R. S. E.

[In addition to its hypnotic effects, chloral is a powerful antiseptic, and may be used for a variety of purposes, such as preservation of anatomical preparations, the injection of bodies for the dissecting-room, and for the dressing of wounds. The following are the conclusions arrived at with regard to its value for dissecting-room purposes.]

1. Bodies injected with the hydrate of chloral are preserved from decay equally well as when the ordinary preservative fluids are used.
2. If exposed to the air, or carelessly attended to by the students, the tissues become very black, and give off a disagreeable, mawkish odor.
3. Delicate nerve-plexuses can be more successfully dissected out when the bodies are injected with chloral solution; and,
4. It is much cheaper than other preservative fluids.

[A solution of five grains of chloral to the ounce of water preserves anatomical preparations better than spirit.]

*As a dressing for wounds and ulcers.*—I have tried chloral extensively as an external application to wounds and abraded surfaces. I found as the result of these experiments that a lotion containing from five to fifteen grains of the hydrate of chloral to the ounce of water formed an excellent dressing to ulcers and wounds, dressed with lint and gutta-percha in the ordinary man ner. I could relate several cases, but I will select only one. A young lad, T. M., lately one of the boys in the Mars training ship, had one of his legs severely burned, and after being treated by the surgeon in charge of the ship for three months, the boy was recommended to go into Dundee hospital to have the limb amputated below the knee. As T. M. was within a fortnight of receiving his discharge from the ship, he was allowed to visit his friends in Edinburg, who were unwilling that the leg should be amputated, and put the boy under my care. I saw him in April last, and found a large ulcer on the leg, extending from a little below the knee to the middle of the foot, and several inches in breadth. The edges were very irregular, and a considerable amount of fœtid discharge came from the ulcer. The boy and his friends were very anxious that I would try and preserve the limb. I ordered a lotion containing fifteen grains of the hydrate of chloral to the ounce of water, some chloral lint, such as is manufactured by J. P. McFarlan & Co., of this city, as recommended by Dr. P. H. Watson, and some gutta-percha, and gave instructions for having the limb dressed twice daily with these. I may mention that previously it had been dressed with some preparation of carbolic acid. After the chloral dressing the limb healed rapidly. The ulcer got gradually less, the fœtid discharge disappeared; and when I saw the patient in July last, only a small ulcer remained, and even that was gradually diminishing in size, notwithstanding the fact that the boy was daily employed in a large drapery shop in town, and was unable to give the leg that rest which was necessary, and which I had so much recommended. The boy is now a sailor, and is at present on a voyage to Athens. I also used chloral solution as an injection into the sacs of large abscesses, and found that it tended much to diminish secretion and make the parts heal. I found it also a useful lotion for the eye in inflammatory conditions of that organ. It is an excellent application to burns, and very specially where there is a fœtid discharge. I also found it a good application to remove warts from the hands and fingers. I used for this purpose a lotion containing 15 to 20 grains to the ounce of water, applied by means of lint and gutta-percha. It causes no pain, and the wart speedily becomes smaller, and gradually disappears.

I also used it as a lotion to sore nipples and to inflamed mucous membranes. When chloral is applied to an ulcer, a wound, or the interior of an abscess sac, it causes at first some smarting, but that only lasts for a few minutes, and is soon succeeded by a most agreeable sensation. Patients so treated have frequently told me that soon after the lotion was applied a very agreeable soothing effect was felt in the wound. I believe that in all such cases chloral acts as a local sedative. It produces anæsthesia of the nerves of the part. Wherever there is a wound or ulcer there is irritability of the nerves of that part; and chloral, by soothing this irritability of the nerves, favors the healing process.

I have frequently used with good effect an ointment containing 30 to 60 grains of the hydrate of chloral to the ounce in eczema, and other allied affections. I believe it to be one of the best applications in such diseases; and a medical practitioner lately told me that he had used it with marked benefit as a local application during an attack of erysipelas of the head. Chloral in various forms has been extensively used in the Royal Infirmary of this city by Dr. P. H. Watson, senior surgeon of that institution, and I have the honor of appending a letter from him giving an account of his experience of hydrate of chloral as a dressing to wounds:

"I have in my wards made use of the chloral hydrate for fully six months, and find it quite as active as an antiseptic as carbolic acid or boracic acid. It approaches nearer to carbolic acid in its effects than to the boracic acid, especially in that it is volatile, and thus by its vapor penetrates and surrounds parts to which as a dressing it has been applied with an atmosphere of itself.

"It has marked advantage over carbolic acid, in so far that its odor is pleasant, resembling some of the ethereal compounds employed for flavoring purposes. It also is absorbed, and, in being so, deadens pain after an operation.

"I employ it in four forms:

"1. A lotion of 5 to 40 per cent. in water, for cleansing away discharges around a wound, cleansing sponges used in operations, and analogous purposes.

"2. An ointment composed of concrete paraffin, white wax (Scotch), and almond oil, to which 1-12th to 1-8th of chloral is added, while the other ingredients are liquefied by heat. The components of the ointment should at once be rubbed together, covered, to prevent the evaporation of the chloral, and cooled to a concrete form as rapidly as may be. It is afterwards rubbed up with a few drops of the solution of chloral to disintegrate it, and prevent its crystalline form being reassumed. This ointment takes great pains to make efficiently. If not properly prepared, it is either inert, containing sometimes, I find, absolutely no chloral; at other times (if made cold) the chloral is so imperfectly mingled that it acts as an irritant, and blisters tender cutaneous surfaces. The ointment is applied spread into the substance of linen cloth, so as to be incorporated with the material. This dressing forms the immediate application to the surface around the wound, and covers in the wound itself. It does not adhere, but peels off like a thin layer of wax.

"3. An external excipient dressing is made by soaking lint in a solution of chloral (dr. i. ad oz. j.). It is then wrung out of this and carefully dried. The care is necessary to avoid long exposure or a high temperature, as this volatilizes the chloral.

"4. Lint soaked in a solution of chloral in olive-oil (1-8) employed to fill cavities such as those left in some excisions, and to employ as compresses when it is desired to prevent bleeding from the cut surfaces in operations for the removal of *dead* bone.

"In some cases, when the chloral appears to act as an irritant, even when carefully prepared, it may be necessary to interpose some impermeable material between the line of operation and the dressing.

"I have never met with any disagreeable results from the absorption of the chloral. On the contrary, I have found the pain of recent wounds only satisfactorially modified and relieved by its employment."—*Edin. Med Journal, Feb.* 1876.

